# Choosing the right tool: Leveraging of plant genetic resources in wheat (*Triticum aestivum* L.) benefits from selection of a suitable genomic prediction model

**DOI:** 10.1007/s00122-022-04227-4

**Published:** 2022-10-01

**Authors:** Marcel O. Berkner, Albert W. Schulthess, Yusheng Zhao, Yong Jiang, Markus Oppermann, Jochen C. Reif

**Affiliations:** Leibniz Institute of Plant Genetics and Crop Plant Research (IPK), 06466 Stadt Seeland, Germany

## Abstract

**Key message:**

Genomic prediction of genebank accessions benefits from the consideration of additive-by-additive epistasis and subpopulation-specific marker effects.

**Abstract:**

Wheat (*Triticum aestivum* L.) and other species of the *Triticum* genus are well represented in genebank collections worldwide. The substantial genetic diversity harbored by more than 850,000 accessions can be explored for their potential use in modern plant breeding. Characterization of these large number of accessions is constrained by the required resources, and this fact limits their use so far. This limitation might be overcome by engaging genomic prediction. The present study compared ten different genomic prediction approaches to the prediction of four traits, namely flowering time, plant height, thousand grain weight, and yellow rust resistance, in a diverse set of 7745 accession samples from Germany’s Federal ex situ genebank at the Leibniz Institute of Plant Genetics and Crop Plant Research in Gatersleben. Approaches were evaluated based on prediction ability and robustness to the confounding influence of strong population structure. The authors propose the wide application of extended genomic best linear unbiased prediction due to the observed benefit of incorporating additive-by-additive epistasis. General and subpopulation-specific additive ridge regression best linear unbiased prediction, which accounts for subpopulation-specific marker-effects, was shown to be a good option if contrasting clusters are encountered in the analyzed collection. The presented findings reaffirm that the trait’s genetic architecture as well as the composition and relatedness of the training set and test set are major driving factors for the accuracy of genomic prediction.

**Supplementary Information:**

The online version contains supplementary material available at 10.1007/s00122-022-04227-4.

## Introduction

Initial endeavors to systematically collect seed samples of landraces of crop plant species were already made more than 100 years ago (Vavilov 1997). These efforts laid the foundation for ex situ conservation of crop plant diversity in genebanks worldwide. Until today, these collections of plant genetic resources (PGR) were steadily increasing to about 7.4 million accessions of historical cultivars, landraces, and wild relatives of crop plant species (FAO 2010). Globally, the *Triticum* genus accounts for more than 11 percent of all preserved accessions and thus, wheat (*Triticum aestivum* L.) and its closely related species outnumber all other plant species (FAO 2010). Such a large share of *Triticum* accessions implies a broad coverage of the biodiversity existing in wheat as well as of the wide adaptation to various climatic conditions, which was acquired in at least 10,000 years of cultivation and breeding (Salamini et al. 2002; Pont et al. 2019). Moreover, these large number undoubtedly also reflects the vital importance of wheat for human nutrition (OECD/FAO 2021). These broad collections of wheat PGR and the mentioned genetic diversity could offer plenty of opportunity to identify donors with beneficial alleles, which are yet undeployed in elite breeding programs. It has always been proposed that leveraging the potential of genebank collections will facilitate the continuous success in breeding to sustain an increasing world population in a changing climate. This aspect gains importance since the germplasm pool in wheat breeding has diminished in genetic diversity (Reif et al. 2005; Pont et al. 2019) due to several decades of continuous and strong selection (Fu and Somers 2009) which was accompanied by a reduction in effective population size in wheat cultivars (Joukhadar et al. 2017). This erosion of genetic diversity in breeding populations was conjectured to be one of the reasons for stagnation in breeding gains (Venske et al. 2019). In stark contrast, studies on PGR report a limited scope of exploitation in breeding so far (Tanksley and McCouch 1997; Mascher et al. 2019). Other authors highlighted mainly the breeding efforts on targeted introgression of specific genes and allele mining for an improvement in qualitative traits (Sharma et al. 2021). However, PGR have scarcely been considered for an informed and targeted application as crossing partners in order to recover narrow breeding populations and to enrich genepools with diverse allelic variation. Among multiple obstacles regarding the exploitation of wheat PGR, insufficient or lacking phenotypic information with respect to economically meaningful traits was the major restriction preventing a wide and informed use of PGR in pre-breeding programs (McCouch et al. 2013; Dempewolf et al. 2017; He et al. 2022).

The large number of present accessions limits the systematic and holistic evaluation of entire wheat collections in field experiments and thus, quantity and quality of the available phenotypic data per accession face strong limitations. For most wheat accessions, phenotypic data were either generated in separate experiments of limited scope or when accessions were propagated in order to refill seed stocks (Mascher et al. 2019). In the latter case, phenotyping was restricted to high-heritable traits such as plant height (PH), flowering time (FT), or thousand grain weight (TGW). Based on the wheat collection of the Federal ex situ Genebank of Agricultural and Horticultural Crops hosted by the Leibniz Institute of Plant Genetics and Crop Plant Research in Gatersleben (IPK Genebank), Philipp and collaborators (2019) recently demonstrated the possibility to integrate historic data of multiple regeneration cycles despite its complex non-orthogonal structure by calculating best linear unbiased estimates (BLUE). The IPK Genebank characterized 12,754 wheat accessions based on historic data for FT, PH and TGW (Philipp et al. 2019). Nevertheless, 36% of the wheat accessions at the IPK Genebank still lack BLUE for these rather standard traits and consequently, their true potential for these traits still remains unknown to research and breeding.

In recent years, genomic-based approaches were widely proposed to predict or verify the phenotype of PGR (Yu et al. 2016; Crossa et al. 2017; Mascher et al. 2019; Gonzalez et al. 2021; Jiang et al. 2021). The underlying rationale of proposing these approaches was the tremendous decrease in the cost for genotyping in recent years; with some authors reporting even a change by more than 100,000-fold in two decades (Akdemir and Isidro-Sánchez 2019). On the other hand, costs for phenotyping have not changed much in the respective timeframe (Akdemir and Isidro-Sánchez 2019). Therefore, implementation of robust procedures of genomic prediction might populate genebank catalogues with phenotypic estimates in a rapid and cost-efficient manner (Yu et al. 2016; Gonzalez et al. 2021; Jiang et al. 2021; Schulthess et al. 2021). A wide range of prediction approaches has been presented (Jannink et al. 2010); however, their suitability and a proof-of-concept for large collections of wheat PGR needs to be examined. Such assessment should mainly focus on the reliability of the predicted phenotypic data since it is a potential drawback of the approach. Knowing all this information, genomic prediction can bridge the gap between ex situ conservation of genetic diversity and its purposeful exploitation in breeding.

All genomic prediction models share a common basic concept with different assumptions about the parameters. Models deduce an assumed association between genotype and trait based on a set of genotypes with known genotypic and phenotypic information. This set of genotypes is referred to as training set (Akdemir and Isidro-Sánchez 2019). The information is applied to predict phenotypic values for genotypes that just have genotypic but no phenotypic information. This set of genotypes is called the test set (Akdemir and Isidro-Sánchez 2019). On closer examination, it nevertheless appears obvious that considerable differences among models lie in assumed characteristics of the set of genotypes or of the trait such as the genetic architecture. While some approaches assume the validity of the infinitesimal model, such as genomic best linear unbiased prediction (G-BLUP) (VanRaden 2008), other models assign more weight to a limited number of markers due to the assumed presence of few major quantitative trait loci, for instance weighted best linear unbiased prediction (W-BLUP) (Bernardo 2014; Zhao et al. 2014). For other approaches, assumptions are that markers have different effect on the phenotype depending on their genetic background, meaning the presence of epistasis or depending on other characteristic of the genotype such as populations structure or breeding type. Examples for models with these specific assumptions are extended genomic best linear unbiased prediction (EG-BLUP) (Jiang and Reif 2015) and general and subpopulation-specific additive ridge regression best linear unbiased prediction (GSA-RRBLUP) (Li et al. 2017), respectively. Considering the contrasting assumptions of the models, we hypothesized that the suitability of the respective approach is trait-specific and depends on the composition of the collection under investigation.

The main objective of this investigation was to evaluate different genomic prediction approaches to fill the above-explained gaps and shortcomings in characterization of PGR based on the example of four quantitative traits, namely FT, PH, TGW and yellow rust resistance (YR). More specifically, we aimed to (1) compare ten genomic prediction approaches, accounting for differences in the trait’s genetic architecture, based on the prediction ability for the four traits, (2) investigate the population structure within the analyzed set of PGR as well as its influence on the prediction, and (3) predict missing phenotypic data for all accession samples based on the most suitable approach for the respective trait.

## Material and methods

### Plant material, phenotypic data and genomic data

The present study was conducted on a diverse set of 7651 accessions which are part of the *Triticum* collection of the IPK Genebank. The selected set of accessions has recently been published by Schulthess and collaborators (2021), who performed the selection of accessions with the intentions to exclusively incorporate winter wheat (*Triticum aestivum* L.). For nine accessions, the taxonomic affiliation has been updated during the conductance of the aforementioned research. While one accession was revealed to be a wheat interspecific hybrid, another eight accessions belonged to different species of the *Triticum* genus. Nevertheless, these accessions were still included in the present study, since this problem can be expected to occur in genebank genomics. Passport data about the origin of all 7,651 accessions were used as it has been presented by Schulthess and collaborators (2021).

The 7651 genebank accessions were used to generate 7745 distinguishable accession samples (Schulthess et al. 2021). Briefly, every accession was grown in a single-row plot in the first year in order to select a single ear of one plant, which represented the dominant phenotype. The selected ear was isolated by bagging before flowering. In few cases, two contrasting morphotypes occurred per accession and thus, each morphotype was considered for isolation with one ear of one representative plant. In the following year, seeds of the selected ears were propagated in an ear-to-row fashion.

Phenotypic data of the traits FT, PH, TGW, and YR, incorporated as BLUE, were derived from two different sources. BLUE for the traits FT, PH, and TGW were used as published by Philipp and collaborators (2019). As described in the aforementioned study, these BLUE were derived from curation and analysis of historical phenotypic data which was gathered during seed regeneration in the IPK Genebank. The number of available BLUE differed between accessions due to the origin of the data and the associated non-orthogonal structure of seed regeneration. While the aforementioned study presented BLUE per accession, the current study relied on developed accession samples and thus, we proceed on the assumption that the BLUE are equally valid for the accession and the respective accession sample. If two accession samples were generated from one accession, both were represented with the same BLUE value. While BLUE values for FT were present for 4593 accession samples, BLUE values for PH and TGW were only present for 4,564 accession samples and 4280 accession samples, respectively (SFig.1). BLUE for the trait YR, which were present for 6300 accessions samples, were retrieved from Schulthess and collaborators (2021). These BLUE values originated from a series of twelve recently executed field trials relying on naturally occurring yellow rust infection. According to Philipp and collaborators (2019), FT, PH, and TGW were recorded in days after the 1^st^ of January, cm (including awns), and g, respectively, while YR levels were reported according to an official 1 (absence of disease symptoms) to 9 (fully infected plants) scoring scale (Schulthess et al. 2021).

Genotyping-by-sequencing profiles of all 7745 accession samples were recently published by Schulthess and collaborators (2021). Concisely, DNA was extracted from seedlings by applying a silica-membrane technology, parallelly digested with two restriction enzymes and ligated to adapters with barcode sequences. Sequencing engaged either an Illumina Hiseq-2500 or a NovaSeq 6000 system. After sequencing, reads were trimmed to 30 bp and aligned to the sequence of the reference genome var. Chinese Spring v1.0 (IWGSC 2018) for SNP calling. Marker information was trimmed based on the homozygous calls for both major and minor allele. Later, SNP variants with > 10% missing values, < 10 homozygous genotypes for each allele, or > 1% heterozygosity were discarded. Missing data were subsequently imputed based on the dominant allele, and markers were afterward re-filtered for minor allele frequencies ≥ 1%. After all these steps, 17,118 SNP variants were retained in the final marker matrix used for downstream analyses.

### Analysis of population structure

The population structure of the examined PGR collection was analyzed in a two-step approach. First, the pairwise Rogers’ distances (Rogers 1972) were calculated based on the genomic data. Second, principal coordinate (PCo) analysis (Gower 1966) was performed on the pairwise Rogers’ distances and the first PCo was plotted against the second and third PCo, respectively. Subsequently, the number of ancestral populations was more precisely determined by using the R package LEA (Frichot and François 2015). Based on the result of the PCo analysis, the possible range of ancestral populations was already assumed to be rather low. Thus, the “snmf” function was applied for up to 20 ancestral populations, *k* ranging from 1 to 20, and under the model assumption that the hexaploid wheat could be treated as diploid. This model was run with 200 iterations and with 100 independent repetitions. The fit of the solved models was evaluated based on the cross-entropy criterion. For all genotypes, admixture between subpopulations was retrieved engaging the “Q” function of the LEA package. For the sake of simplicity, all accession samples were assigned to subpopulations based on the respective ancestral population with the highest admixture coefficient.

The Euclidean distance between the genotypes was calculated on the BLUE for the four traits following the approach described by Sneath and Sokal (1973). In order to avoid any distortion due to the incomplete data structure, Euclidean distances were only calculated for the subset of 3,921 accession samples which have BLUE for the four traits treated in this study.

### Genomic prediction approaches

Ten genomic prediction approaches were assessed in the present study. The most basic genomic prediction approach tested in the current study was G-BLUP (VanRaden 2008). The model accounts for additive genetic effects which are explained by the relationship among the n genotypes included in the set,

1 $$y = 1_{n} \mu + g + e,$$

where $y$ is an *n*-dimensional vector of BLUE for the trait of interest, ${1}_{n}$ represents an n-dimensional vector of ones and $\mu$ denotes the overall mean of the set. $g$ and $e$ are n-dimensional vectors of additive genotypic values and residuals, respectively. Both are considered as random parameters having multivariate normal distribution $\mathrm{g }\sim \mathrm{ N}(0,G{\sigma }_{g}^{2}$) and $\mathrm{e }\sim \mathrm{ N}(0,I{\sigma }_{e}^{2}$). The covariance matrix $G$ is modeled by the additive genomic relationship matrix, which was computed following the first method described by VanRaden (2008), $I$ is an n-dimensional identity matrix, ${\sigma }_{g}^{2}$ is the genetic variance and ${\sigma }_{e}^{2}$ is the residual variance.

EG-BLUP can be considered as an extension of the G-BLUP model which additionally accounts for additive-by-additive epistatic effects (Jiang and Reif 2015). The model has the form (Henderson 1985):

2 $$y = 1_{n} \mu + g + g_{1} + e,$$

where $y$, ${1}_{n}$, $\mu$, $g$, and $e$ are as described above. In addition to (1), the n-dimensional vector ${g}_{1}$ is a random parameter which accounts for additive-by-additive effects between genotypes with ${g}_{1} \sim \mathrm{ N}(0,H{\sigma }_{{g}_{1}}^{2}$). $H$ is the epistatic genomic relationship matrix which is approximately calculated as $H=G\#G$ where “$\#$” denotes the Hadamard product (Jiang and Reif 2015; Martini et al. 2016).

W-BLUP is another derivation of the G-BLUP model which includes associated genetic markers as additional random or fixed parameters (Bernardo 2014; Zhao et al. 2014). These markers were identified with a genome-wide association study which was performed with the R package rrBLUP. The significance of the found associations was tested based on a significance level of 5% alongside engaging the simpleM method (Gao et al. 2008) in order to account for multiple testing. Thereafter, further analysis addressed the impact of the significantly associated markers on the trait. ASReml-R (Butler et al. 2018) was used to estimate the proportion of the overall phenotypic variance explained by each significantly associated marker. The W-BLUP model described by (Bernardo 2014; Zhao et al. 2014) has the form

3 $$y = 1_{n} \mu + g + F_{G} a_{F} + e$$

where $g$ is the n-dimensional vectors of additive genotypic values with $\mathrm{g }\sim \mathrm{ N}(0,{G}_{r}{\sigma }_{g}^{2}$). ${G}_{r}$ is similarly computed as above; however, the included set of markers was reduced by the *r* markers defined based on the genome-wide association study. While ${F}_{G}$ is a n × r matrix which codes for the allele content of the reference allele at a certain locus, ${{\varvec{a}}}_{{\varvec{F}}}$ is the associated vector of additive effects assumed as fixed in the present study. Four different settings of W-BLUP were tested in the current study; they only differed in the approach of defining the set of  *r* markers, denoted by $S$. Firstly, $S$ comprised one marker with the strongest association even if the association was not statistically significant. Secondly, $S$ was the one marker among all significantly associated markers which explained most of the trait’s variance. Thirdly, the significantly associated markers were first sorted according to their explained phenotypic variance in a descending order. Then, $S$ included the first *r* markers which together explained at least 10% of the phenotypic variance and *r* is the smallest number with this property. Fourthly, all significantly associated markers were considered.

GSA-RRBLUP is based on the assumption that the effect of a marker differs depending on the genotype and more precisely, that effects are more similar between genotypes belonging to the same subpopulation (Li et al. 2017). Subpopulation were defined as described earlier, and thus, every genotype had reported admixture coefficients for the different subpopulations as well as one subpopulation with the strongest affiliation. Four different settings of GSA-RRBLUP were tested in the present study. The first two models only considered the genotype’s affiliation to one of the three subpopulations or five subpopulations, respectively $k=3\, or \, 5$. These models had the form,

4 $$y = 1_{n} \mu + Z_{A} a + Z_{{S_{1} }} a_{{S_{1} }} + \ldots + Z_{{S_{k} }} a_{{S_{k} }} + e\, \left( {k = 3\, or\, 5} \right),$$

where $Z$ is a n × m matrix which codes for the allele content of the reference allele, where m is the total number of markers, and $a$ is the m-dimensional vector of additive random effects of the m markers. ${Z}_{{S}_{k}}$ is a matrix, similar to ${Z}_{A}$, which codes for the allele content of the reference allele in those genotypes which were assigned to the ${k}^{th}$ subpopulation while the remaining genotypes were represented here with 0. ${a}_{{S}_{k}}$ is the m-dimensional vector of additive random marker effects specific in the ${k}^{th}$ subpopulation.

In contrast to the above two models, the remaining two accounted for admixture between the three, and five subpopulations, respectively:

5 $$y = 1_{n} \mu + Z_{A} a + Z_{{Sadm_{1} }} a_{{Sadm_{1} }} + \ldots + Z_{{Sadm_{k} }} a_{{Sadm_{k} }} + e\, \left( {k = 3\, or \,5} \right)$$

${Z}_{{Sadm}_{k}}$ is a n × m matrix derived from ${Z}_{A}$ by element-wise multiplication with an n-dimensional vector of admixture coefficients for the ${k}^{th}$ subpopulation, and ${a}_{{Sadm}_{k}}$ is the m-dimensional vector of related random effects especially valid in the ${k}^{th}$ subpopulation.

G-BLUP, EG-BLUP, and GSA-RRBLUP were additionally compared in two scenarios, which involve different subsets of accession samples. Each subset comprised only those two subpopulations which were most contrasting based on the Rodgers’ distances and Euclidean distances, respectively.

### Cross-validation and evaluation of genomic prediction

The genomic prediction approaches were evaluated based on the prediction ability in a fivefold cross-validation. Briefly, all genotypes with BLUE were randomly divided into five equally sized parts of which four parts were assigned to the training set while the test set comprised of the remaining part. Before prediction, the BLUE of the test set have been masked and therefore, prediction relied exclusively on the training set. The procedure was continued until each of the five part had once been assigned to the test set. In order to calculate the prediction ability, the predicted values for the five test sets were combined and compared with the BLUE. The resulting Pearson correlation coefficient equals the prediction ability. This procedure was repeated 100 times independently for the four traits. Per trait, the different genomic prediction models were tested based on the same 100 subdivisions of genotypes in order to avoid any bias. For W-BLUP, the genome-wide association study was performed within each training set of the respective cross-validation. For GSA-RRBLUP, the assignment of genotypes to subpopulation was performed only once with the entire set because it did not use any phenotypic data and hence, would not affect the validity of cross-validation.

For the comparison of the two most contrasting subpopulation, all accession samples of both contrasting subpopulations were included even though the subpopulations might be different in size. This is a more realistic representation of the conditions in genebank collections. Additionally, the prediction models were tested with an equal number of accession samples from the two subpopulations in order to avoid any bias of different sizes. The equal size was obtained by random selection of accession samples from the larger subpopulation. In both cases, the assignment to the five equally sized parts of one fivefold cross-validation was done independently per subpopulation and subsequently, corresponding parts were combined. This strategy aimed to minimize bias in sampling from the two subpopulations.

The entire computational work was performed in the R environment engaging R version 4.0.2. The R code for all prediction models was published in the e!DAL online repository (Arend et al. 2014).

## Results

### Population structure showed strong admixture between subpopulations

**Fig. 1:**
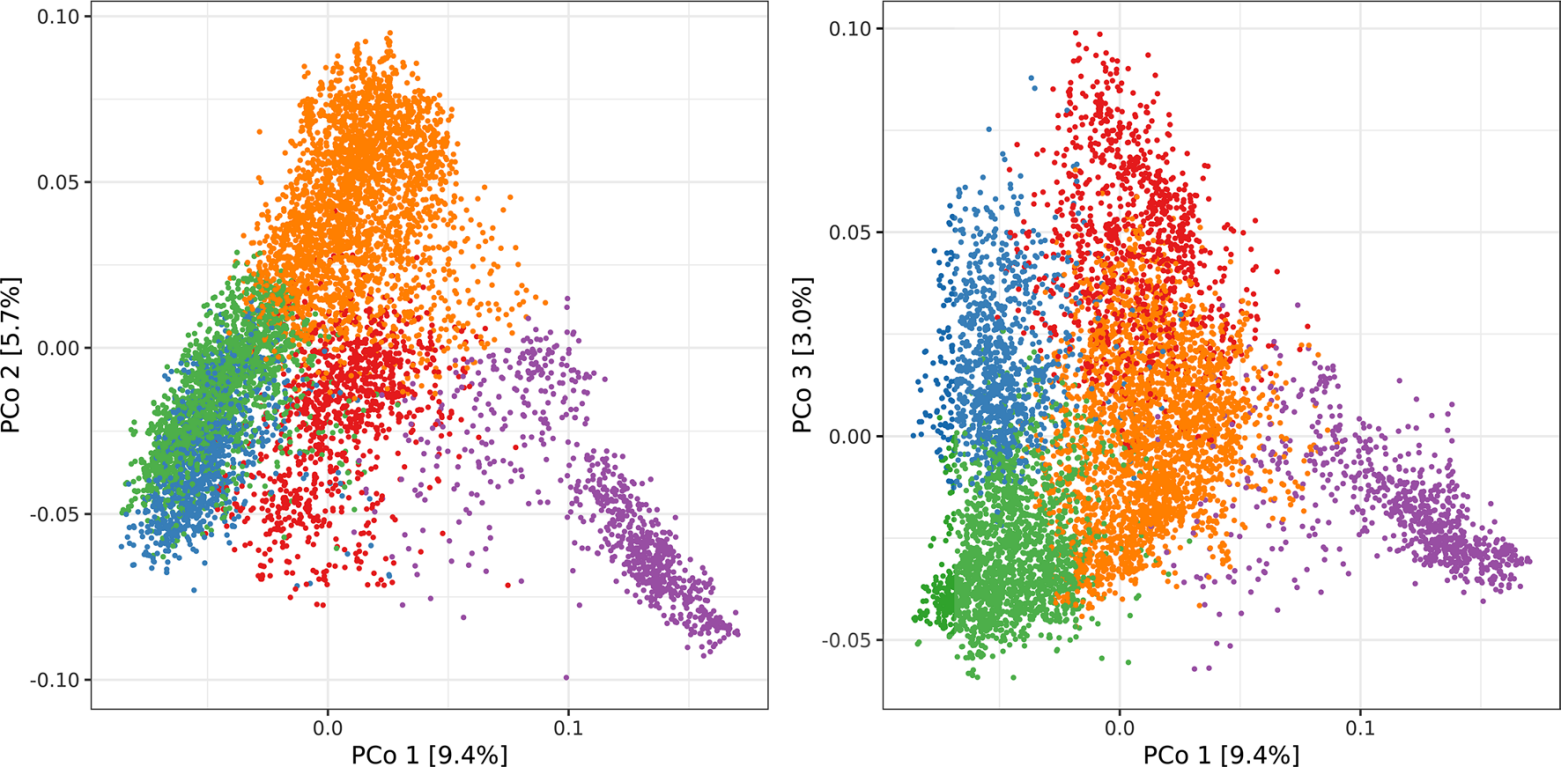
Results of the principal coordinate (PCo) analysis based on the Rogers’ distances. Plots show the second and third PCo, respectively, plotted against the first PCo. Colors mark the association of accession samples with one of the five subpopulations with following color-code: red: Southern European Subpopulation, blue: Western European Subpopulation, green: Central European Subpopulation, purple: Asian Subpopulation, orange: Eastern European Subpopulation. The numbers between brackets indicate the percentage of variation explained by the different PCo

**Fig. 2:**
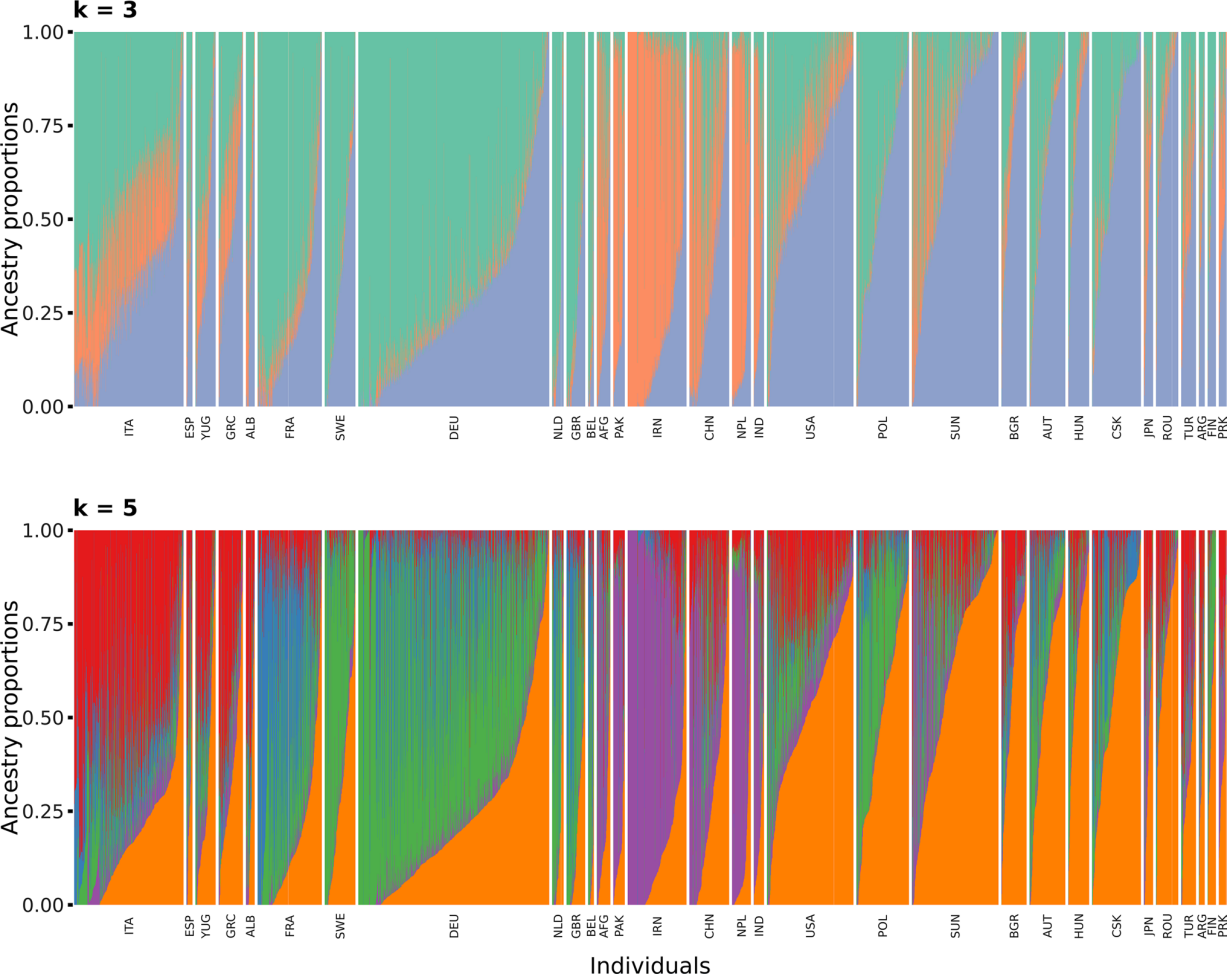
Ancestry proportions of 6225 genotypes displayed separately per country of origin. While the upper plot depicts the scenario involving the presence of three subpopulations (*k* = 3), the lower plot shows the ancestry proportions of the scenario of the presence of five subpopulations (*k* = 5). With respect to all 7745 accession samples, these plots do not display those accession samples which originate from countries with less than 20 associated accession samples (139 accession samples from 25 countries) as well as those from unknown origin (1381 accession samples)

Two different analyses were performed in order to obtain an overview of the genetic diversity in the panel of 7745 accession samples. The results for the first three PCo of the Roger’s distance matrix are depicted in Fig. 1. The majority of the accession samples were shown to be part of one cluster while one smaller subgroup of accession samples could easily be distinguished based on the first PCo. Based on the cross-entropy criterion (SFig. 2), the most explanatory numbers of ancestral populations ranged from three to five. Due to this reason, two different scenarios were followed throughout this study: one scenario assuming three ancestral populations and the other involving five ancestral populations. For both scenarios, admixture coefficients were investigated in comparison with other accession samples originating from the same country (Fig. 2). The main result in this respect was that the vast majority of the accessions samples could not unconditionally be assigned to a single ancestral population, but the analyzed collection was rather strongly affected by admixture of several ancestral populations.

Ancestral populations were clearly associated with a geographic origin. In both scenarios, the most distinct ancestral population was dominantly represented by accession samples which derived from Asian countries such as Iran, China, Nepal, and Pakistan. These accessions samples coincide with those of the aforementioned outgroup which was torn apart by the first PCo. Thus, these accessions were genetically most distinct from the majority of other genotypes. All other accession samples were mainly of European origin or were at least genetically similar to these accession samples. Assuming the presence of five ancestral populations, the four remaining ancestral populations were dominant in accessions samples originating from countries in Southern Europe (such as Italy, Greece, former Yugoslavia, and Albania), Western Europe (mainly France), Central Europe (such as Germany and Sweden) and Eastern Europe (such as the former Soviet Union, former Czechoslovakia, and Austria). It is particularly worth a mention that the latter group also included most accessions which originated from the USA. Accession samples from some countries were, however, well represented in two ancestral populations. As an example, some accession samples from Poland were assigned to the ancestral population from Central Europe, while many others were assigned to the ancestral population from Eastern Europe. Subpopulations were named according to the predominate region of origin (Southern European, Western European, Central European, Asian, and Eastern European). For both scenarios, subpopulations were not equal in size (SFig. 3). This structure of accession samples was not only apparent based on the genotype but was also reflected in the distribution of phenotypic characteristics which were dominant in the respective subpopulation (SFig. 4).

### Quality of prediction enhanced by EG-BLUP and GSA-RRBLUP

**Fig. 3:**
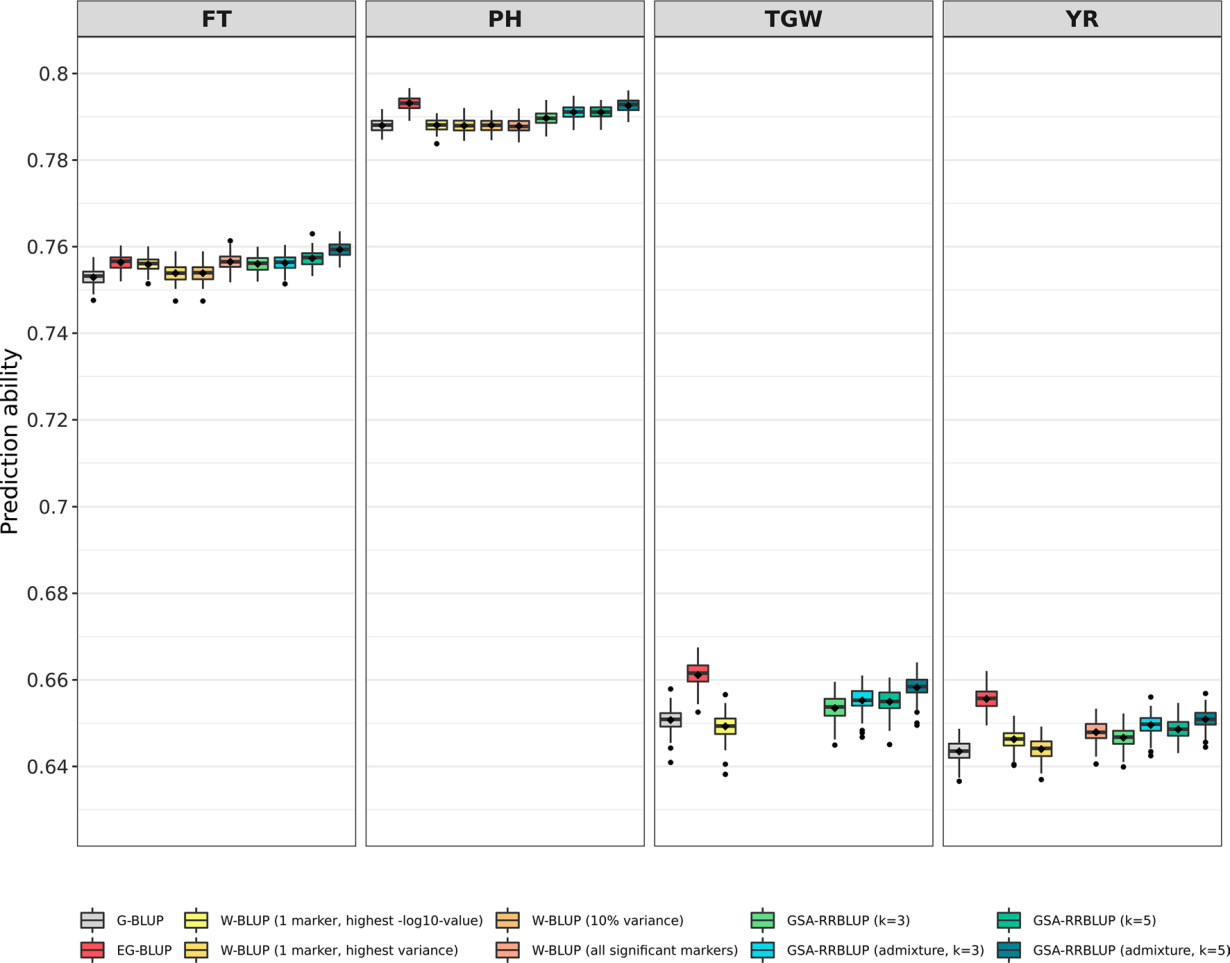
Prediction abilities for ten different genomic prediction models applied on the traits flowering time (FT), plant height (PH), thousand grain weight (TGW), and yellow rust resistance (YR). The prediction abilities were calculated as the correlations between observed and predicted trait performance using 100 complete runs of fivefold cross-validation

Ten genomic prediction approaches were evaluated based on their prediction abilities for the traits FT, PH, TGW, and YR. While large differences in the prediction ability were encountered between traits regardless of the applied model, differences between models for the prediction of one specific trait were less pronounced (Fig. 3). FT was most accurately predicted by the application of GSA-RRBLUP with the incorporation of admixture and the assumed presence of five subpopulations. The prediction ability of this prediction approach was found to be 0.85% higher compared with G-BLUP, the widely used standard model. In addition, both GSA-RRBLUP approaches for five subpopulations outperformed all other approaches; however, accounting for admixture leads to a slight increase in prediction ability by 0.002 (STab. 1). Contrastingly, EG-BLUP outperformed all alternative models concerning the prediction for the remaining three traits. In more detail, the prediction abilities of EG-BLUP exceeded those of G-BLUP for PH, TGW, and YR by 0.65%, 1.6%, and 1.89%, respectively. The prediction ability of EG-BLUP surmounted those of GSA-RRBLUP by 0.06%, 0.44% and 0.72% for the traits PH, TGW, and YR. The preconditions of each of the four W-BLUP approaches were not met for TGW and YR. For the latter trait, only three of the four W-BLUP strategies could be evaluated (Fig. 3) because the sum of all significant markers did not always account for at least 10% of the variation in the trait. For TGW, the absence of significant marker trait association was found to be a strong limitation to the implementation of most W-BLUP approaches. Due to this fact, the performance of only one W-BLUP approach could be assessed for TGW (Fig. 3).

### EG-BLUP with less advantage when facing pronounced population structure

Since the composition of genebank collections can strongly be affected by population structure, genomic prediction models were tested with respect to the robustness on two subsets of the collection. These subsets comprised accession samples of only two strongly contrasting subpopulations. The pairwise contrast between the already defined subpopulations was independently evaluated based on the genetic distances which were given by the Rogers’ distances and separately based on the phenotypic distances which were specified by the Euclidean distances. Mutual comparisons of the mean distances between subpopulations are presented in STab. 2. Based on the Rogers’ distances, the Western European subpopulation and the Asian subpopulation were most distinct. While the Central European subpopulation and the Asian subpopulation were most disparate based on the phenotypic distances.

**Fig. 4:**
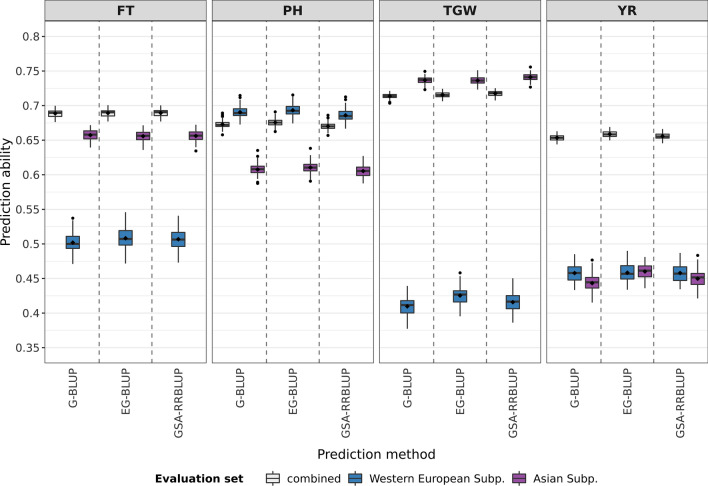
Prediction abilities for three different genomic prediction models applied to the traits flowering time (FT), plant height (PH), thousand grain weight (TGW), and yellow rust resistance (YR) to the accession samples of two subpopulations (Subp.) which are most distinct based on the Rogers’ distances. The prediction abilities were calculated as the correlations between observed and predicted trait performance using 100 complete runs of fivefold cross-validation

**Fig. 5:**
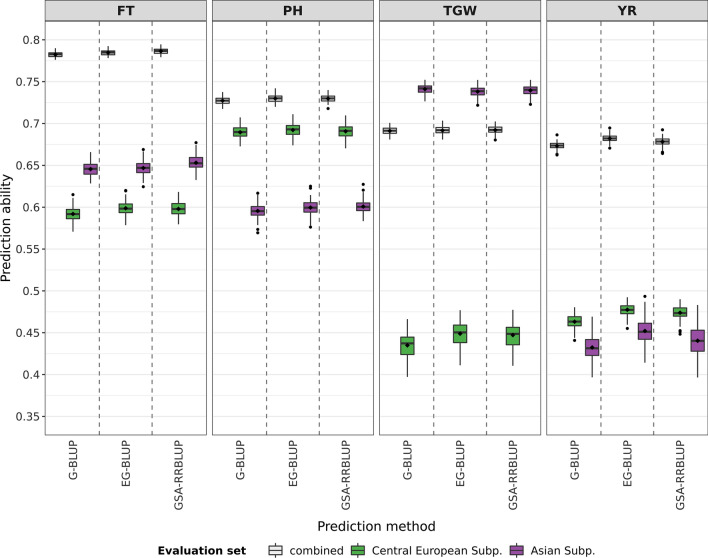
Prediction abilities for three different genomic prediction models applied to the traits flowering time (FT), plant height (PH), thousand grain weight (TGW), and yellow rust resistance (YR) to the accession samples of two subpopulations (Subp.) which are most distinct based on the Euclidean distances calculated from phenotypic information. The prediction abilities were calculated as the correlations between observed and predicted trait performance using 100 complete runs of fivefold cross-validation

Suitability of EG-BLUP and GSA-RRBLUP without an adjustment for admixture was compared within all accession samples of the contrasting subpopulations. G-BLUP was additionally included in the comparison as a well-known standard approach. For the evaluation, prediction abilities were calculated separately for the accession samples of a specific subpopulation as well as for the combined set, as it was done in the previous comparison for the entire collection. Including all accession samples of both subsets, the combined prediction ability was highest for EG-BLUP in the prediction of PH and YR (Figs. 4, 5). For example, EG-BLUP outperformed G-BLUP for the prediction of YR and PH by 1.31% and, respectively, 0.35% in the set with the Central European subpopulation and the Asian subpopulation. In contrast, GSA-RRBLUP showed an overall higher combined prediction ability for the prediction of the traits FT and TGW (Figs. 4, 5). In the set with the Western European subpopulation and the Asian subpopulation, the performance of GSA-RRBLUP exceeded those of G-BLUP for the prediction of FT and TGW by 0.1% and 0.54%, respectively. Therefore, the overall advantage of EG-BLUP which was shown in the previous section could not be proven to be completely resilient in collections with strong population structure. For all traits, the combined prediction ability of the best prediction model differed considerably between both sets of contrasting subpopulations. A difference in prediction ability of 0.0971 was found when applying GSA-RRBLUP for the prediction of FT (STab. 3, STab. 4). Similarly, a difference of 0.0543 was observed for the prediction of PH with EG-BLUP. Differences between both sets of accession samples were by number rather meaningful, while the differences between tested models remained often small. These differences can be considered even more surprising since both sets of subpopulations contained the Asian subpopulation and thus, a large overlap of accession samples was present. A different ranking of genomic prediction models was revealed based on the prediction abilities for each subpopulation. For the Central European subpopulation as well as the Western European subpopulation, EG-BLUP lead to the highest prediction abilities regardless of the trait (Figs. 4, 5). In stark contrast, each prediction model outperformed the remaining two for at least one trait in the Asian subpopulation. For the given dataset, the results indicate that the suitability of genomic prediction models depends on the composition of accession samples.

The three prediction models were additionally tested in a scenario including an equal number of both subpopulations in order to exclude this bias in the training and test sets. For the Western European and the Asian subpopulation, EG-BLUP showed the best performance for the traits PH and YR based on the combined prediction ability and GSA-RRBLUP had the highest combined prediction ability for FT and TGW (SFig. 5, STab. 5). EG-BLUP outperformed the other two models for the prediction of all four traits in the set of the Central European and Asian subpopulation (SFig. 6, STab. 6). Overall, EG-BLUP might thus particularly benefit if biases of the subpopulation’s sizes are excluded. This is, however, not realistic for the application of genomic prediction in genebank collections.

### Populating the genebank catalogue with genomic prediction reveals large diversity

**Fig. 6:**
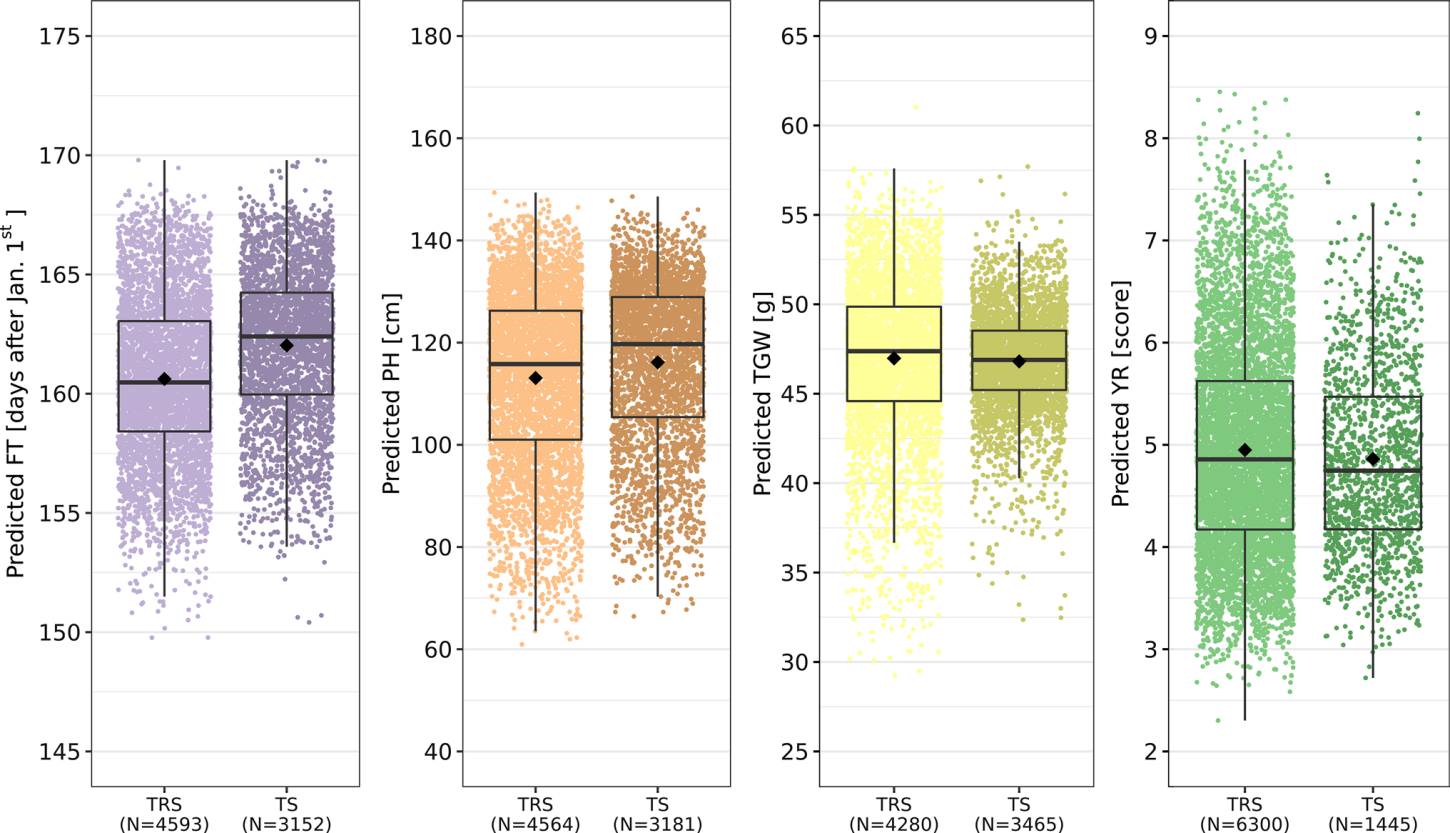
Predictions of flowering time (FT), plant height (PH), thousand grain weight (TGW), and yellow rust resistance (YR) for the entire set of 7745 accession samples. Predictions are depicted separately for accession samples with known phenotypic values, the training set (TRS), and accession samples without phenotypic information regarding the trait of interest which are named test set (TS)

Based on the information of the previously described comparison, the outperforming models of the overall comparison were used in order to predict phenotypic values of the four traits for the entire collection of 7,745 accession samples. While PH, TGW, and YR were predicted with EG-BLUP, FT was predicted with the GSA-RRBLUP considering admixture. In general, genomic predictions revealed the presence of large diversity in all four traits (Fig. 6). Distributions of predicted phenotypes of the training set diverged from the distribution found for the test set with respect to two characteristics: Firstly, variation in the predicted phenotypes was shrunken toward the mean of the test set compared with the training set. This was particularly pronounced for the trait TGW. Secondly, the mean as well as median of the distributions were shifted in the test set in comparison with the training set. While the traits FT and PH were the clearest examples of the latter finding, only minor differences were observed for the traits TGW and YR.

All preditions of the four traits were made publicly available and can be found and downloaded at the search portal for phenotypic data as part of the Genebank Information System of the IPK (https://genebank.ipk-gatersleben.de). These predictions are reported in association with the initial accession, and thus, records of 7563 accessions are enriched with predictions.

## Discussion

Limitations regarding  agronomically relevant information about PGR were identified as major obstacles to the exploitation of PGR as donors in breeding. Genomic approaches have been proposed to surmount this problem, and the aim of the present study is the identification of best practices to predict phenotypic values in diverse collections of wheat PGR. Critical evaluation of the proposed approaches intended to focus on possible obstacles associated with population structures in genebank collections.

### Population structure reflects history of cultivation and breeding

Admixture between different ancestral populations has been shown to strongly affect the analyzed collection of genebank accessions. This finding is in accordance with previous work on the French genebank collection of wheat (Balfourier et al. 2019). The authors pinpointed two main factors driving admixture in wheat. Firstly, the impact of migration processes was concluded to be important for the period before systematic breeding. Secondly, large impact was assigned to the intentional crossing of wheat from different origin in breeding programs. Arguably, both effects account for the admixture, which was observed in the present study. As an illustration, the prominent presence of Eastern European germplasm in accession samples which originated from the USA could be explained by the introduction of Russian and Ukraine germplasm which could endure the continental climate in the former prairies of the USA (Paulsen and Shroyer 2008). Part of this germplasm was brought by immigrants from Eastern Europe in the second half of the nineteenth century (Quisenberry and Reitz 1974), while others introductions can be traced back to targeted expeditions by the United States Department of Agriculture to the Russian Empire (Paulsen and Shroyer 2008). The impact of breeding could for instance be related to the presence of Southern European germplasm in some accession samples from China. More than 30 modern Italian cultivars were used as crossing parent in Chinese breeding programs in the second half of the twentieth century (Zheng 1993) and more than 700 Chinese wheat cultivars were found to originate from these breeding activities (Zheng 1993). In conclusion, the demonstrated admixture represented the diverse history of the worldwide cultivation of wheat which started with domestication some 10,000 years ago (Salamini et al. 2002; Pont et al. 2019) and continues in modern breeding and agriculture.

Despite the pronounced presence of admixture, up to five distinct subpopulations could be identified within the analyzed collection and this distinction had impact on phenotypic differences between subpopulations. The differentiation between the Asian subpopulation and the four European subpopulations, as indicated by the first PCo, agrees with previous analysis of other wheat genebank collections (Balfourier et al. 2019; Pont et al. 2019) and recent results for the IPK wheat genebank using alternative analysis approaches (Schulthess et al. 2021). The fertile crescent, as the center of origin of wheat, forms the nucleus for the spread of wheat toward east and west, respectively (Balfourier et al. 2019; Pont et al. 2019). These opposite directions of dissemination resulted in two main clades in which all European and, respectively, Asian wheat accessions originate. Balfourier and collaborators (2019) drew this conclusion based on a collection exclusively incorporating landraces. In contrast, the analyzed collection of 7,745 accession samples represent not only landraces but also cultivars, which were bred throughout the twentieth century. Therefore, the above described admixture prevented the precise subdivision into subpopulations along regional and cultural borders. This irresolution can be seen in line with previous work on global collections of wheat, which assigned about four fifth of the genetic variation to differences within subpopulations, while differences between subpopulations were consequently found less dominant (Joukhadar et al. 2017). Nevertheless, the distinction into subpopulations can be considered meaningful for the present analysis, since the overall aim was to incorporate this information into genomic prediction. This specific aim was also the motivation to assign all accession samples to the respective subpopulations based on the highest ancestry proportion even if the omission of highly admixed genotypes would often be performed in phylogenetic studies.

### Accounting for additive-by-additive epistasis increases quality of prediction

Ten different genomic prediction approaches were tested for their performance to predict the phenotype in a diverse set of wheat PGR. With cross-validation, the EG-BLUP approach showed the best performance for the prediction of PH, TGW, and YR. The EG-BLUP approach also performed best for the prediction of PH and YR in two subsets of the collection which comprised two contrasting subpopulations. In contrast to the G-BLUP model, EG-BLUP has been shown to account not only for additive effects but also for additive-by-additive epistasis (Jiang and Reif 2015). Therefore, the superiority of EG-BLUP could depend on the genetic architecture of the trait under investigation. The importance of additive-by-additive epistasis was found in many studies on wheat (Martini et al. 2016; Jiang et al. 2017; Raffo et al. 2022) but by extension also for other autogamous plant species such as Arabidopsis (*Arabidopsis thaliana* L.) (Reif et al. 2009) and pigeonpea (*Cajanus cajan* (L.) Millsp.) (Saxena et al. 2021) and furthermore, also in doubled haploid lines of rapeseed (*Brassica napus* L.) (Bocianowski et al. 2017) and maize (Vojgani et al. 2021). Due to the absence of dominance effects in wheat inbred lines, additive-by-additive epistasis is of large importance (Raffo et al. 2022). On the other hand, differences in the genetic architecture might go hand in hand with the selection of genotypes which were included in the training set (Würschum et al. 2017). Raffo and collaborators (2022) concluded that the beneficial effect of the incorporation of additive-by-additive epistasis would depend on the relationship between genotypes of the training and test sets. This cannot be confirmed based on the present genebank collection, which arguably show a low real relationship due to the longtime of diversification accompanied by a break up of haplotype blocks and a reduced linkage disequilibrium. This fact, however, serves as an argument to a contrasting line of reasoning, which does not assign the found benefit of epistatic terms to the genetic architecture of the trait. The epistatic model term can account for purely additive effects if markers are not in complete linkage disequilibrium with the respective causative locus (de los Campos et al. 2019). This illusive epistatic effect has two driving factors. Firstly, the effect increases in importance in larger populations because the interactions between marker can better be detected (de los Campos et al. 2019). Secondly, a relatively low number of markers with a low coverage leads to an overestimation of the epistatic term (Schrauf et al. 2020). Both factors could clearly affect the present results due to the large number of accession samples which were genotyped with genotyping-by-sequencing technology which is known to have lower resolution than other sequencing methods. In conclusion, the benefit of EG-BLUP can neither clearly be assigned to the trait genetic architecture nor revealed as an artifact of the linkage disequilibrium and/or marker coverage. Nevertheless, the model accounting for additive-by-additive epistasis was shown to increase prediction ability and thus, be suitable for application in genebank genomics.

In contrast to EG-BLUP, the importance of the trait’s genetic architecture in the respective training set can clearly be inferred from the obstacles faced regarding W-BLUP. The implementation of some W-BLUP approaches was prevented by the absence of strong marker trait association which account for large variation in TGW and YR, respectively. This finding highlights the pronounced quantitative nature of both traits, and consequently, there are many loci which can explain small portion of the variation in the trait.

### Accounting for population structure may be more beneficial with less admixture

Most genomic prediction approaches assume constancy of marker effects in all genotypes of the training set and test set. Depending on the structure of the analyzed population, this assumption might, however, not be correct due to variation in genetic interactions or in linkage of markers with the respective allele (de los Campos and Sorensen 2014). These variations might most likely attain a maximum in diverse genebank collections in which population structure are more pronounced if compared to, e.g., breeding populations. GSA-RRBLUP was reported to account for subpopulation-specific variation in marker-effects (Li et al. 2017) and with this intention, this model was included in the presented comparison. This approach outperformed all other prediction approaches for the trait FT in the comparisons involving all accession samples as well as both sets of contrasting subpopulations. Different alleles for the same genes for flowering and reproduction could be prevailing in the five subpopulations, and therefore, the ability to assign different effects to each allele might hypothetically cause the advantage of GSA-RRBLUP. Accessions originating from or adapted to regions with different climates and latitudes have evolved or been indirectly selected for different systems to initiate the reproductive phase (Kamran et al. 2014). In their review, Kamran and collaborators (2014) presented a diverse allelic variation for the major gene systems vernalization and photoperiod and further, dominance of some patterns of allelic combinations was reported depending on the region of origin. Additionally, analysis of 410 fairly modern European wheat cultivars has revealed the presence of copy number variations in some major genes which are associated with the variation in flowering time (Langer et al. 2014). Such class of variation cannot be properly displayed by genotyping-by-sequencing markers, and therefore, this allelic variation could only be accounted for by allowing differences in the marker effect. The presence of such variation in a specific group of accession sample remains, however, hypothetical unless otherwise proven. Nevertheless, the genetic architecture of a trait in different subgroups can be pinpointed as a driving factor for the prediction with GSA-RRBLUP.

The presented results indicate that GSA-RRBLUP would have been more effective in the presence of a more pronounced population structure. In contrast to the overall comparison, GSA-RRBLUP showed improved performance relative to EG-BLUP for the reduced set of accession samples with increased genetic and phenotypic distances, respectively. The model could have outperformed EG-BLUP in the overall comparison if the entire collection would have shown a more pronounced structure. Most accessions in the analyzed collection were of European origin, and the Asian subpopulation was the only really distinct outgroup. Studies on other genebank collections indicate that the Asian part of the global diversity in wheat was underrepresented in the analyzed collection (Balfourier et al. 2019; Pont et al. 2019), which was arguably further reinforced by focusing on winter wheat. However, the focus on certain regions of origin will most certainly be present in most genebanks due to the history of collection as well as a focus on national breeding programs. Generally, the choice for GSA-RRBLUP should be made individually based on the prominence of the structure in training set and test set, respectively.

To the best of our knowledge, the present study is the first implementation of GSA-RRBLUP for the prediction of genebank accessions. Moreover, the novelty of incorporation of ancestry proportions as a factor of admixture between different subpopulations was shown to be a promising approach if separation of subpopulations was not strict due to historic exchange or migration with a few examples given above. Both factors, dissemination and exchange as well as separation and clustering, were important factors for many crop species. For instance, rice (*Oryza sativa* L.) has a pronounced population structure with two major subspecies *japonica* and *indica* (Gutaker et al. 2020) and two additional subgroups circumAus and circumBasmati (Santos et al. 2019). Additionally, less dominant admixture has been reported that indicate hybridization events at various timepoints (Choi et al. 2017; Santos et al. 2019). Especially in these cases of alteration in population structure, GSA-RRBLUP accounting for admixture should be further examined and might be promising for populating genebank catalogues.

### Definition of training set plays major role for populating genebank catalogues

We further intended to present the predicted phenotypes for all accession samples as a proof-of-concept for the enrichment of genebank catalogues with predicted phenotypes. A shift in the mean of the predicted phenotypic values as well as a reduction in the variation has been identified between the training and test sets of FT, PH, and TGW. The shift in the mean is associated with different compositions of the training sets. These differences result from the availability of historic data which did not equally represent the different subpopulations. As an example to the contrary, the training set for the prediction of YR was randomly sampled from all accessions samples and thus, hardly any difference in the mean of the predicted phenotypic value was found (SFig. 7).

Characteristics of training sets largely affect the success of prediction which has already been concluded at several places above. Firstly, mean and variance of the phenotypic values in the training set should be approximately representative for the entire collection in order to avoid any bias and extrapolation. These considerations on the training set would thus be trait specific. Secondly, prediction ability is generally positively associated with the size of training set relative to the test set (Edwards et al. 2019). Thirdly, population structure in the test set and training set can compromise the prediction ability (Werner et al. 2020). Under the assumption that subpopulations in genebank collections and crossing families are relatable, one should include as many subpopulations as possible in the training set since this is most beneficial if the size of the training set is fixed (Edwards et al. 2019). Lastly, the genotypes of the training set should be related with those of the test set (Edwards et al. 2019; Werner et al. 2020), which is surely less pronounced in genebank collections than in breeding populations. In conclusion, complications could arise since the most suitable training set should comprise many genetically similar genotypes for every genotype of the test set and in contradiction, the training set should represent all subpopulations and origins of the entire collection with an economically reasonable number of genotypes.

The initial concept of this study builds upon the assumption that the training set would just be defined by the availability of phenotypic data. In some cases, increasing the quality of the training set might leverage the quality of all predicted value and thus, additional phenotyping might, however, be needed. In this respect, targeted selection of genotypes for the training set could be as powerful as the selection of the suitable prediction model and urge for further investigation in the context of genebanks.

### Prospects beyond the explored models

Diverse approaches of genomic prediction have been presented in the past, each having its specific advantages. The ten tested prediction approaches were selected mainly due to the aim of providing a ready-to-use solution which can be implemented by a large group of applicants in diverse applications. Nevertheless, other genomic prediction approaches, such as Bayesian approaches, multiple-trait genomic prediction, and deep learning approaches, could potentially be beneficial.

Bayesian approaches, such as BayesA (Meuwissen et al. 2001), BayesB (Meuwissen et al. 2001), BayesCπ (Habier et al. 2011), and Bayesian LASSO (Legarra et al. 2011), have in common that the genetic variance is partitioned differently for each marker, allowing thus to model different genetic architectures that diverge from the infinitesimal model. The concept, although less flexible, is also used by adjusting main marker effects in W-BLUP. In contrast to the latter approach, Bayesian models do not rely on a two-step analysis involving a genome-wide association study. In a past study on hybrid maize, Li and collaborators (2020) demonstrated that BayesB, BayesC or Bayesian LASSO could only outperform the two-step approach with fixed effects for the prediction of two of in total ten traits. Similarly, W-BLUP showed better performance than BayesCπ for the prediction of heading time and plant height in hybrid wheat (Zhao et al. 2014). Therefore, advantages of Bayesian approaches are trait specific. Although not explored in the current work, the implementation of Bayes approaches in addition to alternative methods could therefore be assessed according to the trait portfolio specific to each genebank.

Multiple-trait genomic prediction can boost the prediction ability of target traits with low heritability provided a genetically correlated indicator trait with higher heritability is included in the model (Calus and Veerkamp 2011; Jia and Jannink 2012). Considering trait correlations (STab. 7) and previously reported heritabilities (Philipp et al. 2019; Schulthess et al. 2021) for PH (0.95), FT (0.92), TGW (0.90), and YR (0.82), the prediction ability for the latter trait could be improved by shifting to multiple-trait prediction using FT or PH as indicator traits. According to trait correlations (STab. 7), taller and/or late flowering wheat plants tend to develop less yellow rust symptoms, which implies that more accurate predictions due to information on PH and/or FT would be more related to “disease escape” than “true” resistance. However, FT and PH have optimal trait values associated with high yields in combination with the specific local agricultural practices (Worland 1996; Austin 1999). Nevertheless, wheat breeders would like to have sources of resistance which are independent of these local requirements. In this context, multiple-trait genomic-prediction models will be rather counterproductive for the informed selection of resistance donors for pre-breeding.

Machine learning approaches can be applied for genomic prediction without many initial assumptions on the genetic architecture of the trait (González-Camacho et al. 2018). Examples of these flexible approaches are artificial neuronal networks and random forest (Azodi et al. 2019). Azodi and collaborators (2019) compared different machine learning models with classical linear approaches on a combination of 18 traits and genomic data from six plant species. While the benefit of random forest was dependent on the dataset, artificial neuronal networks could not outperform the linear approaches (Azodi et al. 2019). Moreover, machine learning models still require much knowledge and computational skills from the applicant and furthermore, they are time-consuming and computationally demanding (Azodi et al. 2019; Montesinos-López et al. 2021). Thus, deep learning will most likely not improve our knowledge of genebank collections right now; nevertheless, future research might reconsider machine learning due to advances in computational efficiency as well as user-friendliness.

### Conclusion

Genomic prediction can be used in order to populate genebank catalogues for wheat in a cost-efficient manner. Furthermore, this approach offers potential for a wide range of crop species which are harbored in genebanks worldwide. Generally, the convincing performance of EG-BLUP and GSA-RRBLUP has been shown based on the prediction of four test traits. We propose the use of EG-BLUP as a standard approach for a wide range of applications in genebank genomics and thus, this model might replace G-BLUP in this respect. On the other hand, GSA-RRBLUP is the preferred choice for more advanced applications if strong population structure is present and elevated computational demand does not impose any restriction. The success of the genomic prediction was shown to be determined by the trait’s genetic architecture and the contrasting or similar composition of the training set compared to the test set. While the first aspect cannot be altered by genebank curators, the latter aspect should receive more attention in future. Targeted selection of genotypes for phenotyping experiments should be considered as a strategy to develop trait-specific training sets. For 7651 accessions, we enriched the publicly available catalogue of the IPK genebank with most accurate predictions of the four examined traits. In the future, predicted data for even complex traits will become available to research and breeding and thus, enable an educated choice of PGR as donors. More than one century after initializing genebank collections, genomic prediction can enable us to understand and activate the accumulated germplasm.

## Supplementary Information

Below is the link to the electronic supplementary material.

SFig. 1 (PNG 1037 kb) Availability of phenotypic and genomic data for the analyzed set of 7,745 accession samples. Numbers shown in association with the circles indicate the number of accessions for the respective overlap of available data. Genotyping-by-sequencing data (GBS) were present for the entire set of accessions, while Best Linear Unbiased Estimations for flowering time (FT), plant height (PH), thousand grain weight (TGW), and yellow rust resistance (YR) were present for subsets.

SFig. 2 (PNG 413 kb) Cross-entropy criterion for a range of 1 to 20 ancestral populations. Whiskers associated with dots depict the standard deviation of the cross-entropy criterion within the 100 repeated runs of the model.

SFig. 3 (PNG 582 kb) Size of subpopulations (Subp.) for the assumed presence of three subpopulations (k=3) and five subpopulations (k=5)

SFig. 4 (PNG 656 kb) Best Linear Unbiased Estimations for the traits flowering time (FT), plant height (PH), thousand grain weight (TGW), and yellow rust resistance (YR). Distributions are shown separately for five subpopulations (Subp.) determined based on the ancestry proportion assuming the presence of five subpopulations.

SFig. 5 (PNG 485 kb) Prediction abilities for three different genomic prediction models applied to the traits flowering time (FT), plant height (PH), thousand grain weight (TGW), and yellow rust resistance (YR) to the accession samples of two subpopulations (Subp.) which are most distinct based on the Rogers’ distances. Both subpopulations were represented by an equal number of accession samples. The prediction abilities were calculated as the correlations between observed and predicted trait performance using 100 complete runs of fivefold cross-validation.

SFig. 6 (PNG 540 kb) Prediction abilities for three different genomic prediction models applied to the traits flowering time (FT), plant height (PH), thousand grain weight (TGW), and yellow rust resistance (YR) to the accession samples of two subpopulations (Subp.) which are most distinct based on the Euclidean distances calculated from phenotypic information. Both subpopulations were represented by an equal number of accession samples. The prediction abilities were calculated as the correlations between observed and predicted trait performance using 100 complete runs of fivefold cross-validation.

SFig. 7 (PNG 16399 kb) Predicted phenotypic values for the traits flowering time (FT), plant height (PH), thousand grain weight (TGW), and yellow rust resistance (YR) shown via color code. Distributions of the predicted values are depicted in the plot of the first and second principal components separately for the training set (TRS) and the test set (TS), respectively.

STab. 1 (DOCX 13 kb) Prediction abilities for the comparison of ten different genomic prediction models applied on the traits flowering time (FT), plant height (PH), thousand grain weight (TGW), and yellow rust resistance (YR). Presented are the mean prediction abilities as well as the associated standard deviation (SD) originating from the 100 complete runs of fivefold cross-validation.

STab. 2 (DOCX 13 kb) Mutual comparison of subpopulations (Subp.) based on the Rogers’ distances and the phenotypic distances, given by the Euclidean distances for the combination of all four traits. Shown are the mean distance between accessions of subpopulation 1 and accessions of subpopulation 2 as well as the associated standard deviation (SD). While Rogers’ distances were calculated based on information on the 7,745 accession samples, Euclidean were only calculated based on Best Linear Unbiased Estimations of 3,921 accession samples with known information for all four traits.

STab. 3 (DOCX 12 kb) Mean and standard deviation (SD) of prediction abilities for three genomic prediction models applied to the genotypes of two subpopulations which are most distinct based on the Rogers’ distances (Western European subpopulation and Asian subpopulation). The prediction abilities were calculated for the traits flowering time (FT), plant height (PH), thousand grain weight (TGW), and yellow rust resistance (YR) as the correlations between observed and predicted trait performance using 100 complete runs of fivefold cross-validation.

STab. 4 (DOCX 12 kb) Mean and standard deviation (SD) of prediction abilities for three genomic prediction models applied to the genotypes of two subpopulations which are most distinct based on the Euclidean distances calculated from phenotypic information (Central European subpopulation and Asian subpopulation). The prediction abilities were calculated for the traits flowering time (FT), plant height (PH), thousand grain weight (TGW), and yellow rust resistance (YR) as the correlations between observed and predicted trait performance using 100 complete runs of fivefold cross-validation.

STab. 5 (DOCX 13 kb) Mean and standard deviation (SD) of prediction abilities for three genomic prediction models applied to the genotypes of two subpopulations which are most distinct based on the Rogers’ distances (Western European subpopulation and Asian subpopulation). Both subpopulations were represented by an equal number of accession samples. The prediction abilities were calculated for the traits flowering time (FT), plant height (PH), thousand grain weight (TGW), and yellow rust resistance (YR) as the correlations between observed and predicted trait performance using 100 complete runs of fivefold cross-validation.

STab. 6 (DOCX 13 kb) Mean and standard deviation (SD) of prediction abilities for three genomic prediction models applied to the genotypes of two subpopulations which are most distinct based on the Euclidean distances calculated from phenotypic information (Central European subpopulation and Asian subpopulation). Both subpopulations were represented by an equal number of accession samples. The prediction abilities were calculated for the traits flowering time (FT), plant height (PH), thousand grain weight (TGW), and yellow rust resistance (YR) as the correlations between observed and predicted trait performance using 100 complete runs of fivefold cross-validation.

STab. 7 (DOCX 12 kb) Correlations between the Best Linear Unbiased Estimations of flowering time (FT), plant height (PH), thousand grain weight (TGW), and yellow rust resistance (YR) in the analyzed set of accession samples.

## Data Availability

All computational work of this study relies on already published data as described in the Material and method section.

## Code availability

The R code of the ten prediction models was published together with the training data for PH, the corresponding subset of the genotypic data, the population structure, and the most accurate predictions for the four traits. The folder with this information can be accessed via the e!DAL online repository (http://dx.doi.org/10.5447/ipk/2022/11).
