# Supplementary figures and images for: Choosing the right tool: Leveraging of plant genetic resources in wheat (Triticum aestivum L.) benefits from selection of a suitable genomic prediction model
Source: Theor Appl Genet. 2022 Oct 1;135(12):4391–407. doi: 10.1007/s00122-022-04227-4 (PMC9734214; doi:10.1007/s00122-022-04227-4)

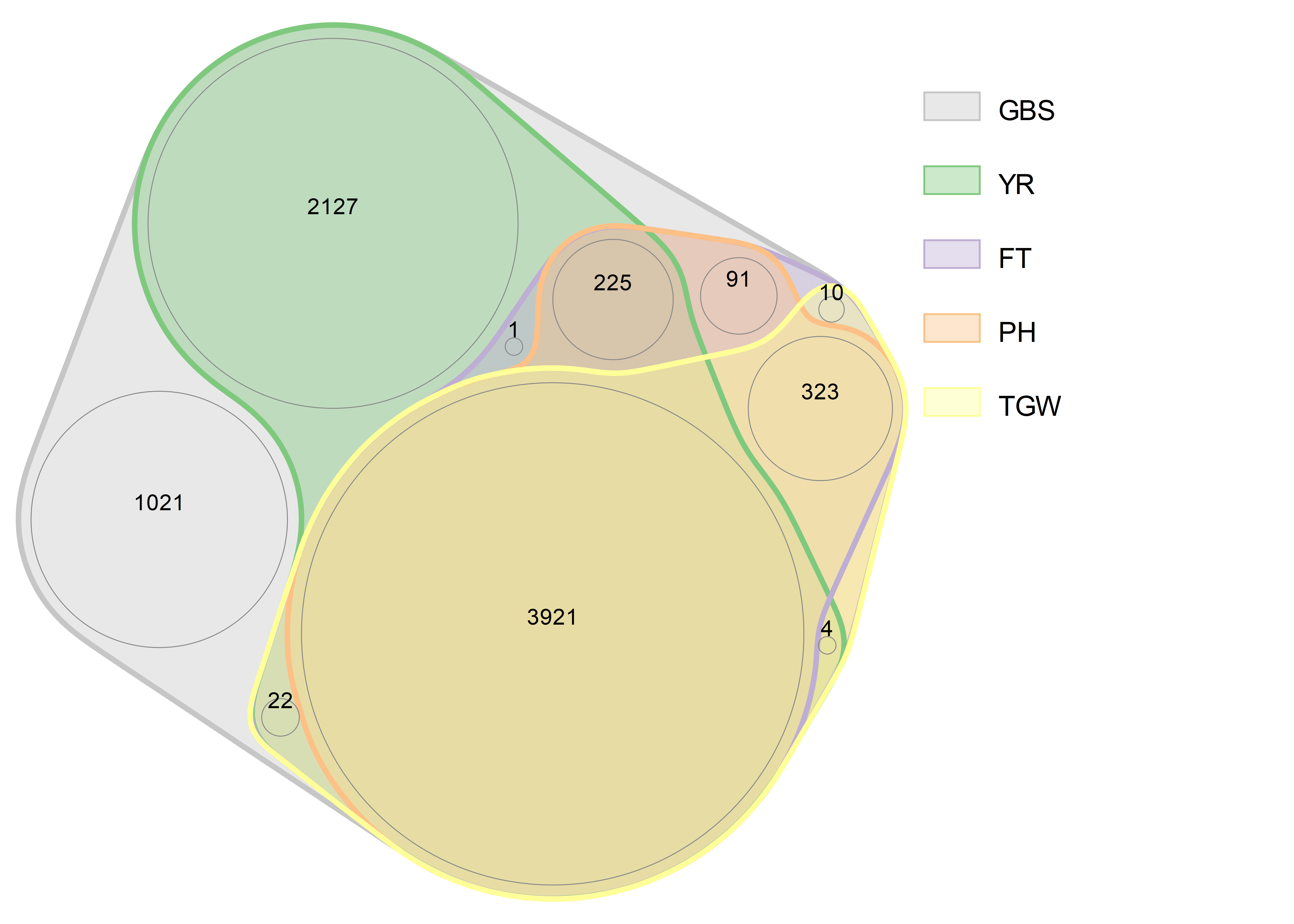

Supplement: Supplementary file 1 — SFig. 1 (PNG 1037 kb) Availability of phenotypic and genomic data for the analyzed set of 7,745 accession samples. Numbers shown in association with the circles indicate the number of accessions for the respective overlap of available data. Genotyping-by-sequencing data (GBS) were present for the entire set of accessions, while Best Linear Unbiased Estimations for flowering time (FT), plant height (PH), thousand grain weight (TGW), and yellow rust resistance (YR) were present for subsets. [file 122_2022_4227_MOESM1_ESM.png]

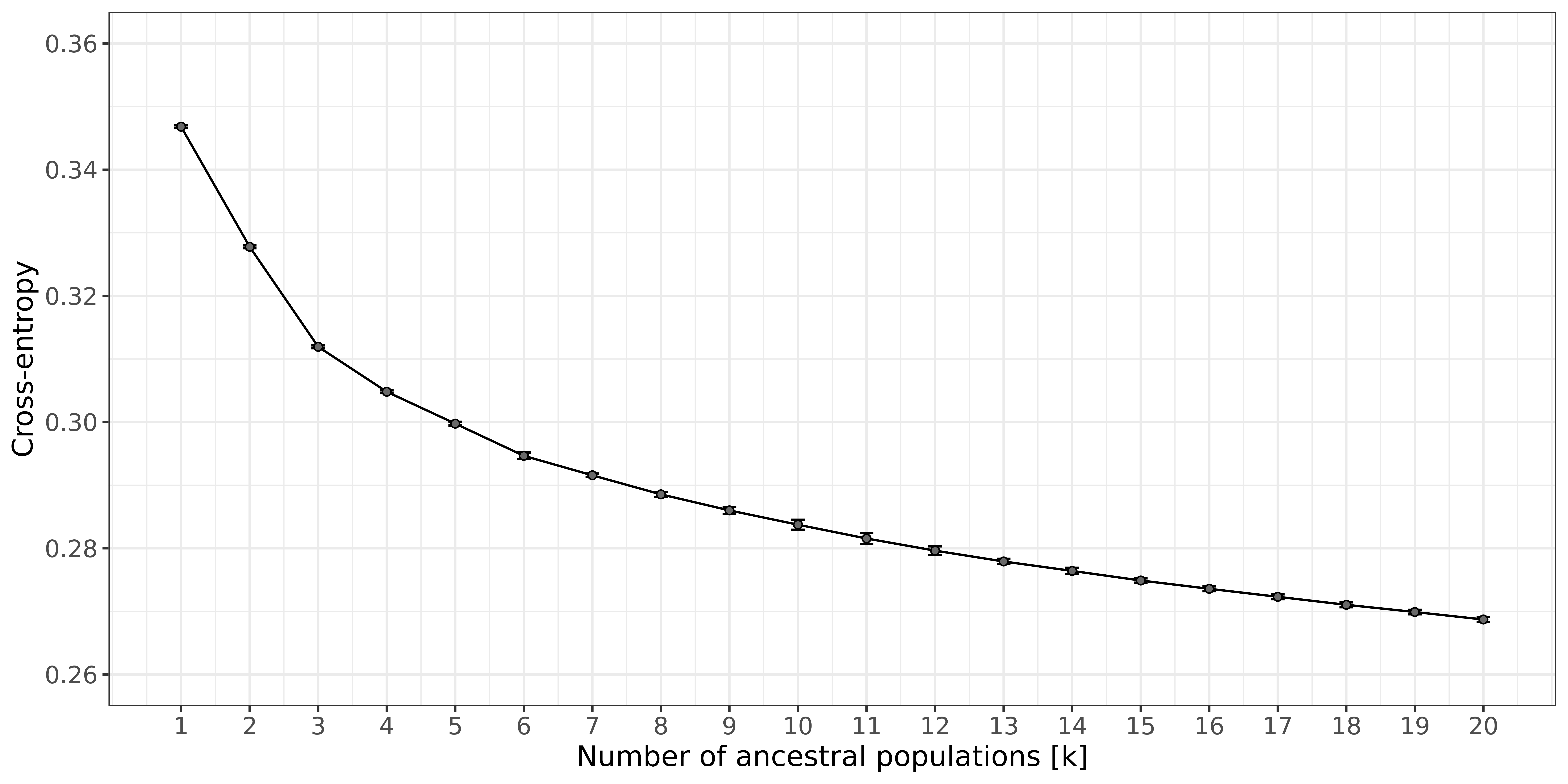

Supplement: Supplementary file 2 — SFig. 2 (PNG 413 kb) Cross-entropy criterion for a range of 1 to 20 ancestral populations. Whiskers associated with dots depict the standard deviation of the cross-entropy criterion within the 100 repeated runs of the model. [file 122_2022_4227_MOESM2_ESM.png]

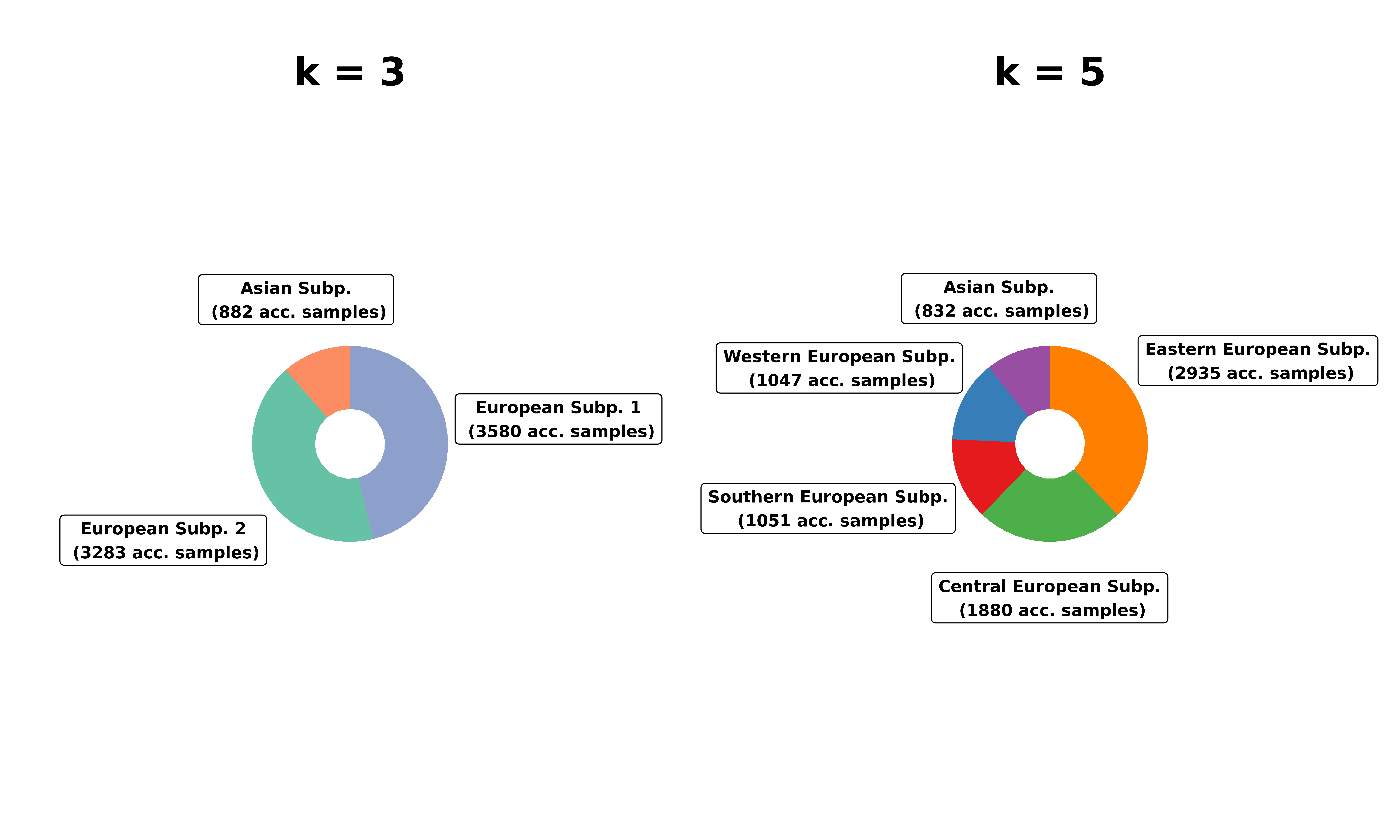

Supplement: Supplementary file 3 — SFig. 3 (PNG 582 kb) Size of subpopulations (Subp.) for the assumed presence of three subpopulations (k=3) and five subpopulations (k=5) [file 122_2022_4227_MOESM3_ESM.png]

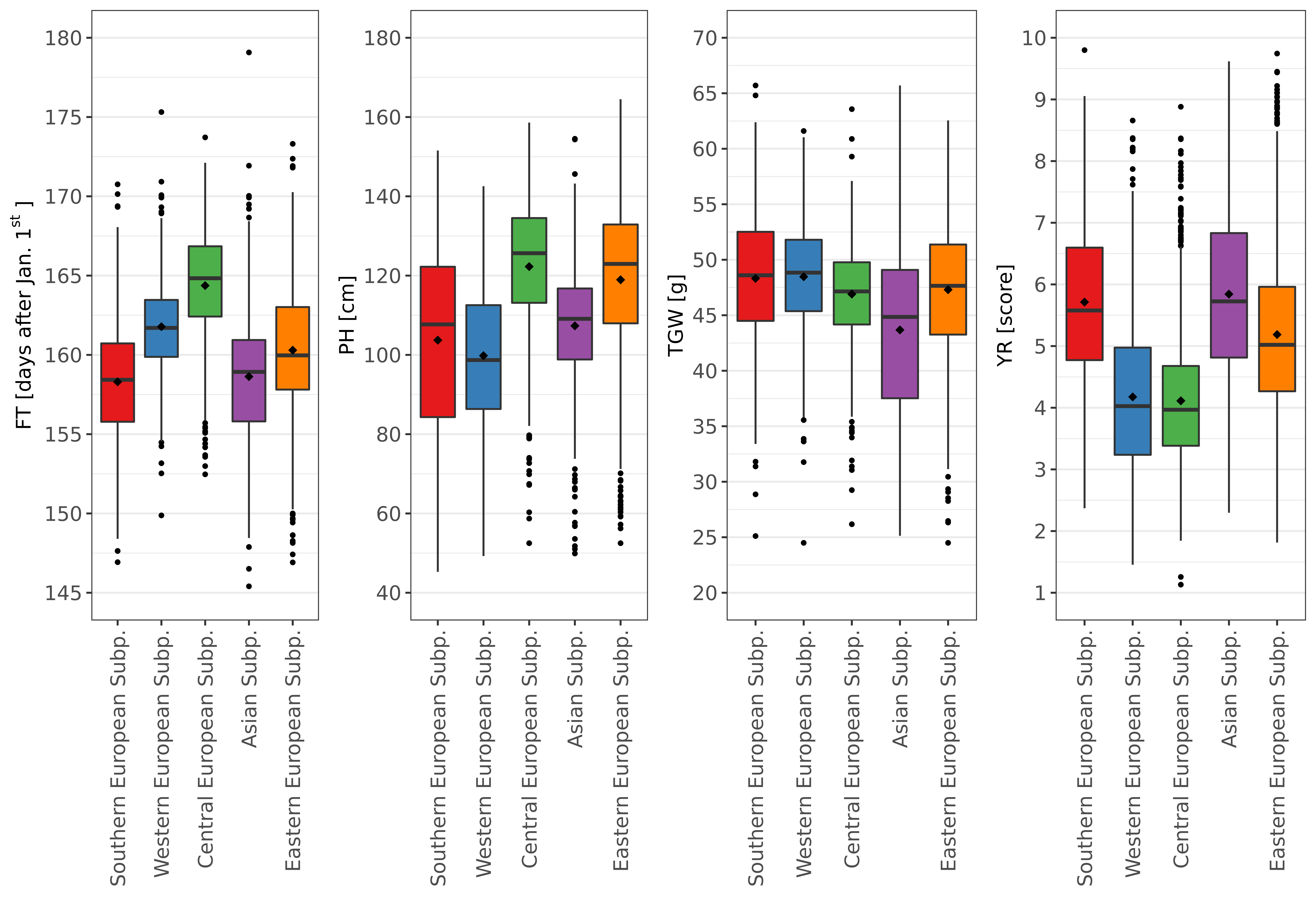

Supplement: Supplementary file 4 — SFig. 4 (PNG 656 kb) Best Linear Unbiased Estimations for the traits flowering time (FT), plant height (PH), thousand grain weight (TGW), and yellow rust resistance (YR). Distributions are shown separately for five subpopulations (Subp.) determined based on the ancestry proportion assuming the presence of five subpopulations. [file 122_2022_4227_MOESM4_ESM.png]

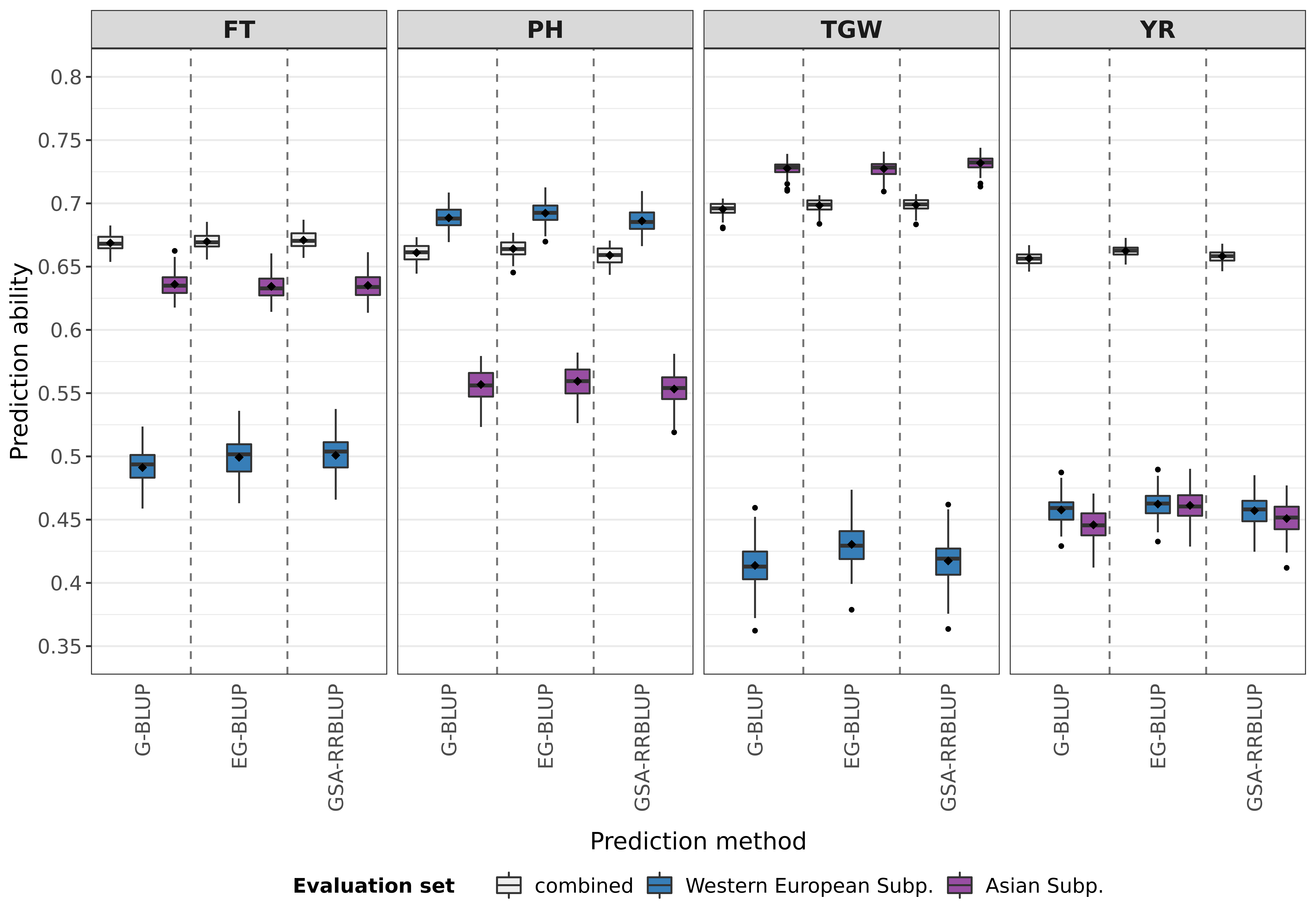

Supplement: Supplementary file 5 — SFig. 5 (PNG 485 kb) Prediction abilities for three different genomic prediction models applied to the traits flowering time (FT), plant height (PH), thousand grain weight (TGW), and yellow rust resistance (YR) to the accession samples of two subpopulations (Subp.) which are most distinct based on the Rogers’ distances. Both subpopulations were represented by an equal number of accession samples. The prediction abilities were calculated as the correlations between observed and predicted trait performance using 100 complete runs of fivefold cross-validation. [file 122_2022_4227_MOESM5_ESM.png]
