# Supplementary material for: Choosing the right tool: Leveraging of plant genetic resources in wheat (Triticum aestivum L.) benefits from selection of a suitable genomic prediction model
Source: Theor Appl Genet. 2022 Oct 1;135(12):4391–407. doi: 10.1007/s00122-022-04227-4 (PMC9734214; doi:10.1007/s00122-022-04227-4)
Supplement: Supplementary file 8 — STab. 1 (DOCX 13 kb) Prediction abilities for the comparison of ten different genomic prediction models applied on the traits flowering time (FT), plant height (PH), thousand grain weight (TGW), and yellow rust resistance (YR). Presented are the mean prediction abilities as well as the associated standard deviation (SD) originating from the 100 complete runs of fivefold cross-validation. [file 122_2022_4227_MOESM8_ESM.docx]

|  | FT | |  | PH | |  | TGW | |  | YR | |
| --- | --- | --- | --- | --- | --- | --- | --- | --- | --- | --- | --- |
|  | Mean | SD |  | Mean | SD |  | Mean | SD |  | Mean | SD |
| G-BLUP | 0.7529 | 0.0018 |  | 0.7880 | 0.0014 |  | 0.6507 | 0.0027 |  | 0.6435 | 0.0024 |
| EG-BLUP | 0.7564 | 0.0018 |  | 0.7931 | 0.0015 |  | 0.6611 | 0.0029 |  | 0.6556 | 0.0024 |
| W-BLUP (1 marker, highest  -log10-value) | 0.7559 | 0.0017 |  | 0.7881 | 0.0014 |  | 0.6493 | 0.0029 |  | 0.6463 | 0.0024 |
| W-BLUP (1 marker, highest variance) | 0.7539 | 0.0019 |  | 0.7879 | 0.0015 |  | - | - |  | 0.6440 | 0.0025 |
| W-BLUP (10% variance) | 0.7539 | 0.0019 |  | 0.7881 | 0.0015 |  | - | - |  | - | - |
| W-BLUP (all significant markers) | 0.7565 | 0.0018 |  | 0.7878 | 0.0016 |  | - | - |  | 0.6479 | 0.0025 |
| GSA-RRBLUP (k=3) | 0.7560 | 0.0018 |  | 0.7897 | 0.0016 |  | 0.6535 | 0.0029 |  | 0.6467 | 0.0023 |
| GSA-RRBLUP (admixture, k=3) | 0.7562 | 0.0018 |  | 0.7911 | 0.0015 |  | 0.6553 | 0.0028 |  | 0.6496 | 0.0023 |
| GSA-RRBLUP (k=5) | 0.7573 | 0.0018 |  | 0.7910 | 0.0017 |  | 0.6550 | 0.0030 |  | 0.6486 | 0.0024 |
| GSA-RRBLUP (admixture, k=5) | 0.7593 | 0.0017 |  | 0.7926 | 0.0016 |  | 0.6582 | 0.0028 |  | 0.6509 | 0.0023 |
|  |  |  |  |  |  |  |  |  |  |  |  |
