# Supplementary material for: Choosing the right tool: Leveraging of plant genetic resources in wheat (Triticum aestivum L.) benefits from selection of a suitable genomic prediction model
Source: Theor Appl Genet. 2022 Oct 1;135(12):4391–407. doi: 10.1007/s00122-022-04227-4 (PMC9734214; doi:10.1007/s00122-022-04227-4)
Supplement: Supplementary file 9 — STab. 2 (DOCX 13 kb) Mutual comparison of subpopulations (Subp.) based on the Rogers’ distances and the phenotypic distances, given by the Euclidean distances for the combination of all four traits. Shown are the mean distance between accessions of subpopulation 1 and accessions of subpopulation 2 as well as the associated standard deviation (SD). While Rogers’ distances were calculated based on information on the 7,745 accession samples, Euclidean were only calculated based on Best Linear Unbiased Estimations of 3,921 accession samples with known information for all four traits. [file 122_2022_4227_MOESM9_ESM.docx]

| Set for comparison | | |  | | Rogers’ dist. | | | |  | | Euclidean dist. | | |
| --- | --- | --- | --- | --- | --- | --- | --- | --- | --- | --- | --- | --- | --- |
| Subpopulation 1 | Subpopulation 2 |  | | Mean | | SD | |  | | Mean | | SD | |
| Southern European Subp. | Western European Subp. |  | | 0.2147 | | 0.0199 | |  | | 2.6401 | | 1.0246 | |
| Southern European Subp. | Central European Subp. |  | | 0.2109 | | 0.0193 | |  | | 3.0446 | | 1.1932 | |
| Southern European Subp. | Asian Subp. |  | | 0.2536 | | 0.0193 | |  | | 2.7150 | | 1.0650 | |
| Southern European Subp. | Eastern European Subp. |  | | 0.2219 | | 0.0175 | |  | | 2.6651 | | 1.1151 | |
| Western European Subp. | Central European Subp. |  | | 0.1767 | | 0.0168 | |  | | 2.4367 | | 0.9186 | |
| Western European Subp. | Asian Subp. |  | | 0.2563 | | 0.0185 | |  | | 2.7954 | | 1.1152 | |
| Western European Subp. | Eastern European Subp. |  | | 0.2076 | | 0.0164 | |  | | 2.5489 | | 0.9452 | |
| Central European Subp. | Asian Subp. |  | | 0.2479 | | 0.0190 | |  | | 3.0452 | | 1.1846 | |
| Central European Subp. | Eastern European Subp. |  | | 0.1945 | | 0.0173 | |  | | 2.5432 | | 1.0301 | |
| Asian Subp. | Eastern European Subp. |  | | 0.2440 | | 0.0181 | |  | | 2.7093 | | 1.1128 | |
|  |  | |  | |  | |  | |  | |  | |  |
