# Supplementary material for: Choosing the right tool: Leveraging of plant genetic resources in wheat (Triticum aestivum L.) benefits from selection of a suitable genomic prediction model
Source: Theor Appl Genet. 2022 Oct 1;135(12):4391–407. doi: 10.1007/s00122-022-04227-4 (PMC9734214; doi:10.1007/s00122-022-04227-4)
Supplement: Supplementary file 13 — STab. 6 (DOCX 13 kb) Mean and standard deviation (SD) of prediction abilities for three genomic prediction models applied to the genotypes of two subpopulations which are most distinct based on the Euclidean distances calculated from phenotypic information (Central European subpopulation and Asian subpopulation). Both subpopulations were represented by an equal number of accession samples. The prediction abilities were calculated for the traits flowering time (FT), plant height (PH), thousand grain weight (TGW), and yellow rust resistance (YR) as the correlations between observed and predicted trait performance using 100 complete runs of fivefold cross-validation. [file 122_2022_4227_MOESM13_ESM.docx]

|  | FT | |  | PH | |  | TGW | |  | YR | |
| --- | --- | --- | --- | --- | --- | --- | --- | --- | --- | --- | --- |
|  | Mean | SD |  | Mean | SD |  | Mean | SD |  | Mean | SD |
| G-BLUP | 0.783 | 0.0029 |  | 0.7214 | 0.0053 |  | 0.6971 | 0.0038 |  | 0.6698 | 0.0042 |
| EG-BLUP | 0.7856 | 0.0027 |  | 0.7244 | 0.0051 |  | 0.6983 | 0.0041 |  | 0.6764 | 0.004 |
| GSA-RRBLUP | 0.7853 | 0.0029 |  | 0.7228 | 0.0051 |  | 0.6981 | 0.0038 |  | 0.6728 | 0.004 |
|  |  |  |  |  |  |  |  |  |  |  |  |
