# Supplementary material for: Choosing the right tool: Leveraging of plant genetic resources in wheat (Triticum aestivum L.) benefits from selection of a suitable genomic prediction model
Source: Theor Appl Genet. 2022 Oct 1;135(12):4391–407. doi: 10.1007/s00122-022-04227-4 (PMC9734214; doi:10.1007/s00122-022-04227-4)
Supplement: Supplementary file 14 — STab. 7 (DOCX 12 kb) Correlations between the Best Linear Unbiased Estimations of flowering time (FT), plant height (PH), thousand grain weight (TGW), and yellow rust resistance (YR) in the analyzed set of accession samples. [file 122_2022_4227_MOESM14_ESM.docx]

|  | PH | TGW | YR |
| --- | --- | --- | --- |
| FT | 0.5055 | 0.0446 | -0.4589 |
| PH | - | 0.1651 | -0.2233 |
| TGW | - | - | -0.0999 |
|  |  |  |  |
